# Being *Aquifex aeolicus*: Untangling a Hyperthermophile’s Checkered Past

**DOI:** 10.1093/gbe/evt195

**Published:** 2013-11-26

**Authors:** Robert J.M. Eveleigh, Conor J. Meehan, John M. Archibald, Robert G. Beiko

**Affiliations:** ^1^Department of Biochemistry and Molecular Biology, Dalhousie University, Halifax, Nova Scotia, Canada; ^2^Faculty of Computer Science, Dalhousie University, Halifax, Nova Scotia, Canada

**Keywords:** *Aquifex aeolicus*, Thermotogae, phylogenomics, hyperthermophiles, lateral gene transfer

## Abstract

Lateral gene transfer (LGT) is an important factor contributing to the evolution of prokaryotic genomes. The Aquificae are a hyperthermophilic bacterial group whose genes show affiliations to many other lineages, including the hyperthermophilic Thermotogae, the Proteobacteria, and the Archaea. Previous phylogenomic analyses focused on *Aquifex aeolicus* identified Thermotogae and Aquificae either as successive early branches or sisters in a rooted bacterial phylogeny, but many phylogenies and cellular traits have suggested a stronger affiliation with the Epsilonproteobacteria. Different scenarios for the evolution of the Aquificae yield different phylogenetic predictions. Here, we outline these scenarios and consider the fit of the available data, including three sequenced Aquificae genomes, to different sets of predictions. Evidence from phylogenetic profiles and trees suggests that the Epsilonproteobacteria have the strongest affinities with the three Aquificae analyzed. However, this pattern is shown by only a minority of encoded proteins, and the Archaea, many lineages of thermophilic bacteria, and members of genus *Clostridium* and class Deltaproteobacteria also show strong connections to the Aquificae. The phylogenetic affiliations of different functional subsystems showed strong biases: Most but not all genes implicated in the core translational apparatus tended to group Aquificae with Thermotogae, whereas a wide range of metabolic and cellular processes strongly supported the link between Aquificae and Epsilonproteobacteria. Depending on which sets of genes are privileged, either Thermotogae or Epsilonproteobacteria is the most plausible adjacent lineage to the Aquificae. Both scenarios require massive sharing of genes to explain the history of this enigmatic group, whose history is further complicated by specific affinities of different members of Aquificae to different partner lineages.

## Introduction

Lateral (or horizontal) gene transfer (LGT) is a potent force in the evolution of cells and their genomes. The evidence is particularly strong in the case of prokaryotes, where the rates of LGT can vary substantially among different lineages. At one end of the spectrum, the genomes of intracellular bacteria such as *Buchnera* (Bordenstein and Reznikoff 2005) and *Rickettsia* (Renesto et al. 2005) display little evidence of LGT, whereas ubiquitous organisms like *Pseudomonas* have dynamic genomes with LGT facilitating their adaption to new habitats (Mathee et al. 2008; Holloway and Beiko 2010). Rampant LGT has led some to reject the idea of a “tree” of prokaryotes in favor of webs or networks (Hilario and Gogarten 1993; Bapteste et al. 2005; Puigbò et al. 2010). The hypothesis of rampant LGT has inspired new models that emphasize LGT as a process contributing to the generation of phylogenetically coherent bacterial groups, as opposed to eroding them (Gogarten et al. 2002; Andam et al*.* 2010).

Different lineages of thermophilic organisms appear to have shared a particularly large number of genes, suggesting that LGT may have played a key role in adaptation to very hot environments (Aravind et al. 1998; Deckert et al. 1998; Nelson et al. 1999; Zhaxybayeva et al. 2009). Members of the genus *Aquifex*, such as *Aquifex aeolicus* VF5, are among the most extreme thermophilic bacteria known, occupying a habitat originally thought to be exclusively occupied by members of the archaeal domain. *Aquifex* lends its name to the order Aquificales and phylum Aquificae, a group based on 16S ribosomal DNA (rDNA) phylogeny (Huber et al. 1992; Cole et al. 2009) with considerable phylogenetic, ecological, morphological, and metabolic diversity, including the freshwater, filamentous *Thermocrinis ruber* (Huber et al. 1998); the acidophile *Hydrogenobaculum acidophilum* (Stohr et al. 2001); and obligate anaerobes in the family Desulfurobacteraceae (L’Haridon et al. 2006).

**Fig. 1.—:**
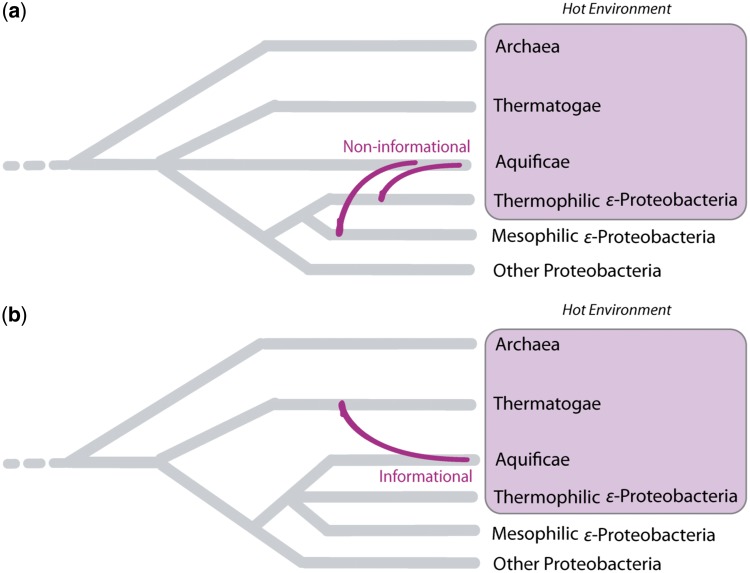
Two alternative hypotheses concerning the closest phylogenetic partners of the Aquificae. (*a*) As suggested by 16S rDNA analysis and some concatenated protein phylogenies, the Aquificae are a deep branching phylum sister to the Thermotogae, with strong affinities for the Epsilonproteobacteria due to large-scale gene sharing. (*b*) The Aquificae are Epsilonproteobacteria or a sister to this group, with extensive exchange of essential genes, either unidirectionally or reciprocally, with other thermophilic lineages such as Thermotogae and Archaea.

Attempts to determine the evolutionary position of the enigmatic Aquificae phylum have usually supported one of two conflicting hypotheses (Huber and Hannig, 2006) (fig. 1): either the Aquificae are basal like the Thermotogae, a phylum containing many hyperthermophiles, and possibly sister to them (fig. 1*a*) or the Epsilonproteobacteria, a diverse class that includes environmental mesophiles, human-associated pathogens, and thermophilic and mesophilic species abundant in hydrothermal habitats (fig. 1*b*; Campbell et al. 2006; Nakagawa et al. 2007). Analyses of widespread or universally distributed informational genes involved in replication, transcription, and translation (Jain et al. 1999) that are believed to be relatively recalcitrant to LGT tend to place Aquificae as an early branch in the tree, in agreement with the 16S rDNA phylogeny. Data sets that supported this conclusion include the reciprocally rooted elongation factor Tu/G (Baldauf et al. 1996), RNA polymerase b/b_0_ chain sequences (Bocchetta et al. 1995), and larger, concatenated alignment-based phylogenies of ribosomal proteins (Wolf et al. 2001; Ciccarelli et al. 2006). However, other studies have contradicted this claim. Beiko et al. (2005) reported a weakly supported *Aquifex + Thermotoga* affiliation (≥0.95 posterior probability [PP] support among only 22 of the 110 constituent protein trees) with a larger number of protein trees supporting *A. aeolicus* as a basal member of the Proteobacteria, a sister to the Epsilonproteobacteria, or a lineage branching within this group. Moreover, phylogenetic profiling corrected for unequal taxon representation identified proteins with *Aquifex + Thermotogae* affinity (including the 22 constituent trees) to frequently co-occur with the Archaea (Aravind et al. 1998; Zhaxybayeva et al. 2009), most notably the Euryarchaeota, suggesting that such proteins may have spread more recently via LGT. The alternative epsilonproteobacterial affiliation was observed among other subsets of informational genes such as the sigma transcription initiation factors (Gruber and Bryant 1998), the *rpoBC* operon (Klenk et al. 1999) and domain architecture studies of *rpoC* (Griffiths and Gupta 2004; Iyer et al. 2004). Biochemical studies of the cytochrome *bc* complex (Schutz et al. 2000) and cell wall characters (Cavalier-Smith 2002) also supported epsilonproteobacterial affiliations.

Based on the predictions of the complexity hypothesis, Boussau, Guéguen et al. 2008) tested the phylogenetic consistency of informational versus noninformational proteins and their respective affiliations, and concluded that the *Aquifex* lineage has strong affinities with the Thermotogae based on the strength of support from informational proteins and a concatenated alignment of nearly universal genes. However, gene trees in which *Aquifex* was sister to heterogeneous groups of organisms were removed, potentially removing a substantial amount of information from the analysis. Although trees based on concatenated alignments in both Boussau, Guéguen et al. 2008 and Wu and Eisen (2008) paired *A. aeolicus* or Aquificae with Thermotogae, the updated tree of Wu et al. (2009), which included the additional Aquificae *Sulfurihydrogenibium* sp. YO3AOP1, moved the phylum into a sister position to the Epsilonproteobacteria (Wu et al. 2009). This discrepancy exists despite the inclusion by Boussau, Guéguen et al. 2008 of *Sulfurihydrogenibium azorense* in their analysis and suggests that different members of phylum Aquificae may show different degrees of affinity to other lineages, and a high degree of sensitivity to the choice of genes used in a concatenated alignment.

In light of these apparently mosaic genomic affinities of the Aquificae, it is unclear which of the competing hypotheses regarding the positioning of phylum Aquificae (fig. 1) is correct; indeed it is unclear whether phylogenomic data can distinguish between these two (and potentially other) alternatives. If the scenario implied by aggregated analysis of informational genes is correct, then the Aquificae are a deep-branching phylum, sister to the Thermotogae, whereas the epsilonproteobacterial, archaeal, and other affinities reflect large-scale highways of gene sharing (Beiko et al. 2005). Alternatively, the Aquificae may be unique Epsilonproteobacteria, either descendants of a thermophilic or mesophilic epsilonproteobacterial ancestor that exchanged essential genes either reciprocally or nonreciprocally with other thermophilic lineages (i.e., Thermotogae and Archaea) due to their common residence in very hot habitats. Thermophilic Epsilonproteobacteria have been identified from hydrothermal habitats using 16S rDNA analysis (Nakagawa et al. 2005; Campbell et al. 2006) and their genomes sequenced: for example, the genomes of the thermal vent Epsilonproteobacteria *Nitratiruptor* sp. SB155-2 (a thermophile) and *Sulfurovum* sp. NBC37-1 (a mesophile) were determined in Nakagawa et al. (2007). If, as suggested by their lifestyle, such organisms are the closest relatives of Aquificae, then their inclusion in genome-level studies should provide vital data in support of this relationship. Boussau, Guéguen et al. 2008 included these genomes in a modified version of their main concatenated analysis, but did not explore their roles as potential bridges between mesophilic Epsilonproteobacteria and the Aquificae in single-gene phylogenies.

Aggregation-based approaches (e.g., trees from concatenated alignments and supertrees) aim to combine signals from many different genes to yield a more-accurate reconstruction of organismal history, and appear to be robust in the face of moderate amounts of missing data (Philippe et al. 2004; Baker et al. 2009; but see Simmons [2011] for demonstrations of increased sensitivity). However, trees based on concatenated alignments are invalid if the concatenated genes have conflicting histories (Leigh et al. 2008). Similarly, supertrees and other combined analyses can be sensitive to incongruence and inclusion of many sequence sets with different evolutionary histories (Bininda-Emonds and Sanderson 2001; Beiko et al. 2008), although Galtier (2007) found considerable resilience of supertrees in the face of high rates of LGT for small numbers of taxa. Here, we assess the phylogenetic placement of Aquificae using two complementary approaches: phylogenetic profiles (Gaasterland and Ragan 1998; Pellegrini et al. 1999), which can inform phylogeny based on the distribution of homologous proteins; and phylogenetic trees, which consider not the distribution but the degree of relatedness of homologous proteins. We apply these approaches to the genomes of three members of phylum Aquificae (comprising *A. aeolicus*, *Hydrogeobaculum* sp. Y04AAS1, and *Sulfurihydrogenibium* sp. YO3AOP1) in light of previously published hypotheses and using a reference set of 774 completely sequenced prokaryotic genomes. The evolutionary history of the Aquificae implicates different partner lineages, most notably the Archaea, Thermotogae, Deltaproteobacteria, and thermophilic members of Nitrospirae, Clostridia, and Epsilonproteobacteria, with different lineages making disproportionate contributions to different molecular subsystems.

## Materials and Methods

### Data Set Acquisition, Homologous Cluster Determination, and Phylogenetic Analysis

A data set comprising 774 genomes, including 721 genomes from 20 bacterial phyla and 53 genomes from four archaeal phyla, was retrieved from the National Center for Biotechnology Information (NCBI) FTP site in December 2008. The set included the genomes of 633 mesophiles, 47 thermophiles, 28 hyperthermophiles, 14 psychrophiles, and 52 with no identified temperature preference. The three Aquificae included in the data set were *A. aeolicus* VF5 (*Aquifex*), *Hydrogenobaculum* sp. Y04AAS1 (*Hydrogenobaculum*), and *Sulfurihydrogenibium* sp. YO3AOP1 (*Sulfurihydrogenibium*), all annotated as hyperthermophiles. The other phyla containing thermophiles or hyperthermophiles are shown in table 1. BlastP version 2.2.19 (Altschul et al. 1997) using a maximum expectation value threshold of 10^−^^3^, and the inclusion of up to 100,000 hits per query, was used to compare the encoded proteins of all three Aquificae genomes against the full set of genomes. Homologous clusters of sequences were defined by first constructing a graph with protein sequences of the three Aquificae as nodes and edges connecting pairs of proteins with bidirectional BlastP expectation values (e values) ≤10^−^^10^. Clusters were then generated by merging all BlastP matches (e values ≤ 10^−^^5^) reported for each connected Aquificae node by using one *Aquifex* node, when applicable, as the seed for a cluster and removing duplicated BlastP matches.

**Table 1:** The Species Distribution of 774 Genomes, 53 Archaea, and 721 Bacteria, Categorized by Domain, Phylum, Class, and Number of Thermophiles Used for Phylogenomic Analysis

	Species/Strains	Thermophiles
**Archaea**	53	31 (58%)
Crenarchaeota		
Thermoprotei	16	15
Euryarchaeota		
Archaeoglobi	1	1
Halobacteria	5	1
Methanobacteria	3	1
Methanococci	7	1
Methanomicrobia	9	1
Methanopyri	1	1
Thermococci	5	5
Thermoplasmata	3	3
Unclassified Euryarchaeota	1	0
Korarchaeota		
Unclassified Korarchaeota	1	1
Nanoarchaeota		
Unclassified Nanoarchaeota	1	1
**Bacteria**	721	44 (6%)
Acidobacteria		
Acidobacteria	1	0
Solibacteres	1	0
Actinobacteria		
Actinobacteria	55	3
Aquificae		
Aquificae	3	3
Bacteroidetes		
Bacteroidia	8	0
Flavobacteria	4	0
Sphingobacteria	2	0
Unclassified Bacteroidetes	1	0
Candidate division TG1		
Unclassified Candidate division	1	0
Chlamydiae		
Chlamydiae	13	0
Chlorobi		
Chlorobia	11	1
Chloroflexi		
Chloroflexi	4	3
Dehalococcoidetes	3	0
Cyanobacteria		
Gloeobacteria	1	0
Unclassified Cyanobacteria	32	3
Deinococcus-Thermus		
Deinococci	4	2
Dictyoglomi		
Dictyoglomia	1	1
Firmicutes		
Bacilli	99	5
Clostridia	37	12
Fusobacteria		
Fusobacteria	1	0
Nitrospirae		
Nitrospira	1	1
Planctomycetes		
Planctomycetacia	1	0
Proteobacteria		
Alphaproteobacteria	93	0
Betaproteobacteria	61	0
Deltaproteobacteria	21	0
Epsilonproteobacteria	22	1
Gammaproteobacteria	190	1
Unclassified Proteobacteria	1	0
Spirochaetes		
Spirochaetes	16	0
Tenericutes		
Mollicutes	23	0
Thermotogae		
Thermotogae	7	7
Verrucomicrobia		
Opitutae	1	0
Unclassified Verrucomicrobia	1	1
Verrucomicrobiae	1	0

A subset of all clusters covered both main candidate partner groups of Aquificae (Thermotogae and Epsilonproteobacteria) and was examined in greater depth. Protein sequences from these clusters were aligned using FSA version 1.15.3 (using fast and maxsn commands: Bradley et al. 2009), then a HMMER profile (version 3.0 using trim command; Eddy 2009) was generated for each FSA alignment. hmmalign outputs a multiple sequence alignment with confidence scores that assess the uncertainty in the alignment; ambiguously aligned regions with a consensus PP threshold less than 0.80 were removed. This trimming procedure discarded approximately 8% (15,118/200,890) of residues from the original protein sequences. To reduce the size of large sequence sets, neighbor-joining phylogenies were inferred using the NEIGHBOR program using a JTT model in the PHYLIP version 3.68 package (Felsenstein 1989): Any genera with more than two represented genomes that constituted a homogeneous clan in the neighbor-joining tree were reduced to two representative sequences, with one representative sampled from each descendant of the earliest implied split in that genus. The sets of retained congener sequences were realigned with FSA + hmmalign as above. Maximum-likelihood phylogenies were inferred using RAxML 7.04 (Stamatakis 2006), using the WAG + Γ (four discrete rate categories) substitution model with 100 rapid bootstrap replicates. We chose the WAG model because it had the best performance, on average, across large data sets in previous validations (Whelan and Goldman 2001; Beiko et al. 2006).

### Testing the Internal Cohesion of Aquificae

Two separate strategies were used to assess the strength of support for the phylogenetic cohesion of the three Aquificae genomes. First, each cluster of proteins was interpreted as a phylogenetic profile. For each profile, a protein *P* from *A. aeolicus* was assigned a rank of 1, and all other proteins in the profile were ranked in ascending order of BlastP expectation value (i.e., in decreasing order of statistical significance) obtained from a comparison using *P* as query and each other protein as subject. If *k* other proteins from phylum Aquificae were present in the profile, then their expected ranking would be 2, 3, … , *k* + 1 if the group were unaffected by LGT involving other phyla: Clusters exhibiting this pattern were termed “clean.” If, however, one or more proteins from members of other phyla were ranked higher than some Aquificae proteins, we termed this set of homologs a “dirty” cluster. Such patterns generally arise due to 1) gene acquisitions by at least one of the Aquificae or non-Aquificae species, 2) a gene duplication event that preceded the divergence of the Aquificae from other lineages, and/or 3) statistical artifacts (Koski and Golding 2001). As we focus on single-copy gene clusters in the Results section, duplication followed by differential loss is unlikely to make a substantial contribution to the inference of phylogenetic partners, although it cannot be completely ruled out. Ranked phylogenetic profiles were complemented with a tree-based assessment of Aquificae using the trees generated with RAxML. In cases where multiple Aquificae genomes were present along with genomes from non-Aquificae lineages, the resulting tree would either contain a single homogeneous clan (Lapointe et al. 2010) with all represented Aquificae genomes and no other genome or a heterogeneous clan in which the cohesion of Aquificae was disrupted by the presence of intruder sequences from other phyla.

### Assessment of Relationships between Aquificae and Other Lineages

The homologous sequence sets were analyzed in terms of their presence/absence distribution across all sequenced genomes (i.e., phylogenetic profiles: Pellegrini et al. 1999). Given our focus on the putative origins of the Aquificae, we considered profiles in which at least one such genome (*Aquifex* = A; *Hydrogenobaculum* = H; *Sulfurihydrogenibium* = S) was represented, and then considered the presence or absence of Archaea (R), Epsilonproteobacteria (E), and Thermotogae (T). Phylogenetic profiles could also be exclusive (designated with ø) to the lineages identified or potentially inclusive (designated with *) of other groups not explicitly named: for example, profiles designated ET-ø have at least one represented protein from Aquificae, Thermotogae, and the Epsilonproteobacteria, and no other lineage, whereas profiles designated ET-* could potentially include representatives from other groups (e.g., other proteobacterial classes or Cyanobacteria) as well.

To identify sets of gene trees supporting particular hypotheses (i.e., a complete Aquificae clan adjacent to E, T, and/or R groups), each of the 100 bootstrap trees generated by RAxML was represented as a set of splits to assess the relative positions of each operational taxonomical unit, with respect to a homogeneous clan of Aquificae. For any pair of taxonomic groups X and Y, the relative support for each of these two groups in association with phylum Aquificae was determined by enumerating the number of bootstrap trees in which group X was closer to the Aquificae (i.e., separated by fewer internal edges) than was group Y, and subtracting the number of trees in which Y was closer to the Aquificae than was X. Replicates in which both X and Y were equidistant to the Aquificae contributed 0 to the total score. The balance of support for X–Y ranged between 100 (all trees support a closer affinity of Aquificae to group X) to −100 if the reverse was true. Thresholding was applied to identify those trees, which have strong preferences for one affinity versus the other: Any tree in which the bootstrap preference for one hypothesis over the other was more than 70% was included in this set.

### Functional Classification of Clusters and System-Level Analysis

All Aquificae gene sets were assigned functions based on the clusters of orthologous groups (COGs; Tatusov et al*.* 1997) classifications, which include 25 specific functional categories grouped into four parent categories using the following approaches: 1) Clusters that contained *A. aeolicus* VF5 were annotated by directly mapping the NCBI locus ID to the associated COG locus ID using the NCBI COG database available at ftp://ftp.ncbi.nih.gov/pub/COG/COG/ (last accessed December 6, 2013). 2) Clusters not assigned in the first step were annotated by determining the most frequent COG annotation among all Blast matches with an e value threshold of 10^−^^15^. 3) Clusters that contained only Aquificae or lacked a defined COG function were assigned Gene Ontology (GO) terms if the evidence codes were experimentally (IMP, IGI, IPI, IDA, or IEP) or computationally (ISS, IGC, or ICA) verified. GO terms were assigned COG functions by using the COG2GO database provided by Gene Ontology (Gene Ontology Consortium 2000). 4) Clusters still lacking a COG annotation were designated as unknown and assigned a functional role of poorly characterized.

Characterizations of biological subsystems/pathways were first performed by identifying general phyletic patterns using the COG designations. For each COG category, a variable preference index (VPI) was computed to contrast the affinities between R, E, and T by expressing the proportion of nonubiquitous profiles that contained inclusive R, E, or T relative to the total number of profiles, excluding lineage-restricted profiles: for example, the VPI for epsilonproteobacterial signal was calculated as (E + RE + ET)/(RET + RE + RT + ET + R + E + T + Other). Comparisons of the VPI values across the 21 COG categories identified specific functional groups in light of the competing Aquificae hypotheses and were corroborated with the identification of KEGG reference pathways.

To identify metabolic pathways and complexes within the COG classification scheme, each Aquificae NCBI RefSeq GI number was mapped to a KEGG orthology (KO) number, which consists of a manually defined, similarity- and positional-based orthologous gene set that corresponds to a node (enzyme or protein) in a specific KEGG pathway (or network; Kanehisa and Goto 2000). For each pathway, a manually drawn reference (denoted by a “ko” number) was constructed to identify the presence/absence of genes within the network of nodes. Metabolic pathways, which were generally widely conserved, were represented with one manually drawn reference pathway from which many organism-specific pathways were computationally generated. Conversely, regulatory pathways were far more divergent and require the construction of separate organism-specific pathways by identifying reference pathways common among groups of organisms (e.g., three ribosomal assembly diagrams for Bacteria, Archaea, and Eukaryota). Each enzyme or protein present in an Aquificae metabolic or regulatory pathway was coupled with manual curation of their associated putatively orthologous cluster and subjected to phylogenetic and bipartition analysis.

To determine whether specific subsets of genes support a particular hypothesis in aggregate, gene groups were clustered into biological subsystems and a supermatrix of all gene alignments was created for each subsystem. Prior to the assessment, sets of in-paralogs were reduced to a single representative by removing all leaf nodes except the one with the shortest branch. Additionally, genes with similar taxonomic distributions were retained for the analysis. A phylogenetic tree was created from each supermatrix using RAxML as mentioned earlier. These concatenation-derived trees were compared with the individual gene trees by first pruning the concatenation-derived tree to the same taxa as the gene tree using DendroPy (Sukumaran and Holder 2010) and then retrieving the per-site log likelihoods of both trees using RAxML based upon the gene alignment and the same model of evolution (WAG + Γ + F). These per-site log likelihoods were compared between the gene tree and concatenation-derived tree using the AU test as implemented in CONSEL version 0.20 (Shimodaira and Hasegawa 2001).

## Results

### Cohesion of the Aquificae

**Fig. 2.—:**
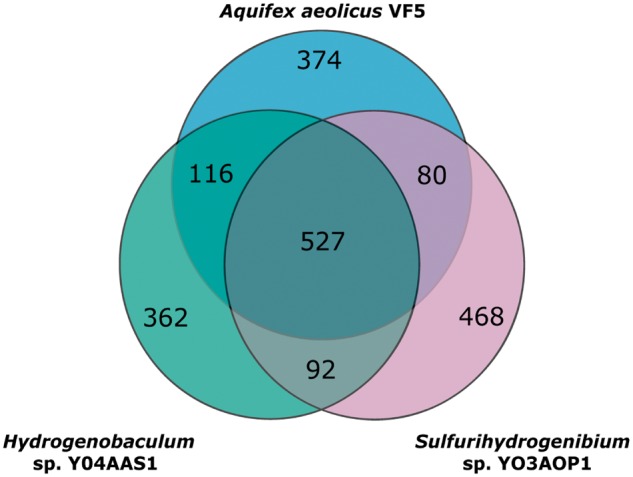
Overlap in homologous gene content among the three sequenced members of phylum Aquificae.

Using the protein-coding genes of the three Aquificae as seeds for the clustering algorithm, 2,295 clusters (2,019 [88%] putatively orthologous, single-copy and 276 [12%] multiple-copy Aquificae clusters) were generated by the extraction of subgraphs defined by reciprocal BlastP matches. Of the 2,019 single-copy Aquificae clusters (fig. 2), 1,204 (60%) were exclusive to one of the three Aquificae (A, H, or S) and 288 (14%) were represented in two Aquificae (AH, AS, and HS). These genes have a patchy distribution due to shared ancestry and subsequent loss, genes invented in specific lineages, and/or LGT. The Aquificae “core” comprising 527 (26%) shared clusters (AHS) could potentially represent genes that were present in a common ancestor and retained in all sampled descendent lineages.

**Fig. 3.—:**
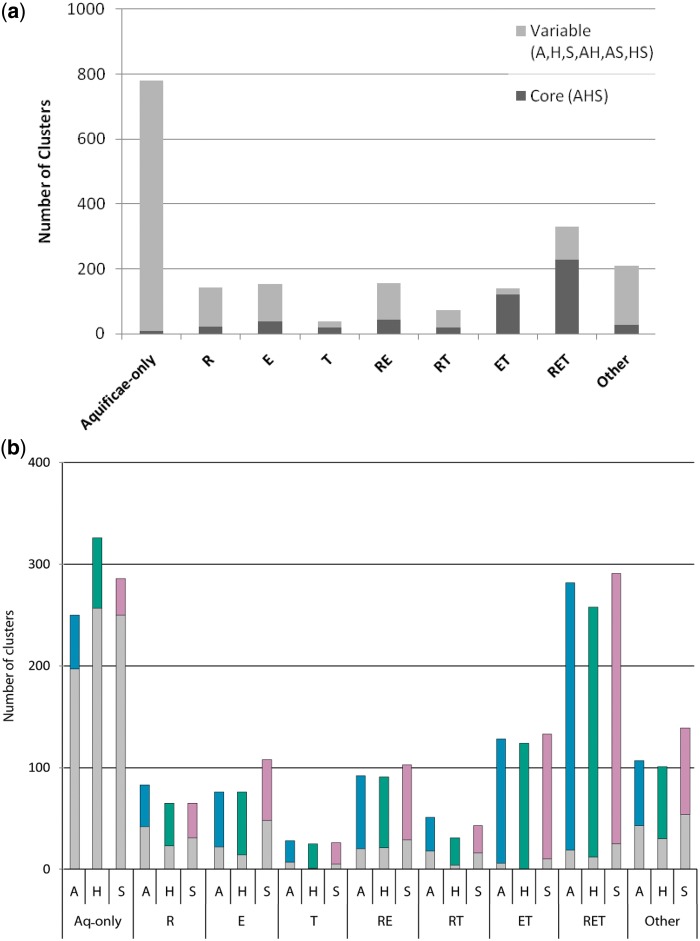
(*a*) Summary of all phylogenetic profiles of all Aquificae subsets, subdivided into the core AHS subset and the variable A, H, S, AH, AS, and HS subsets. (*b*) Phyletic breakdowns for the lineage-restricted subsets (A-only, H-only, and S-only in gray) and inclusive Aquificae subsets (in color) whereby the inclusive *Aquifex* subsets includes AH, AS, and AHS, the inclusive *Hydrogenobaculum* subsets includes AH, HS, and AHS and the inclusive *Sulfurihydrogenibium* subset AS, HS, and AHS.

**Fig. 4.—:**
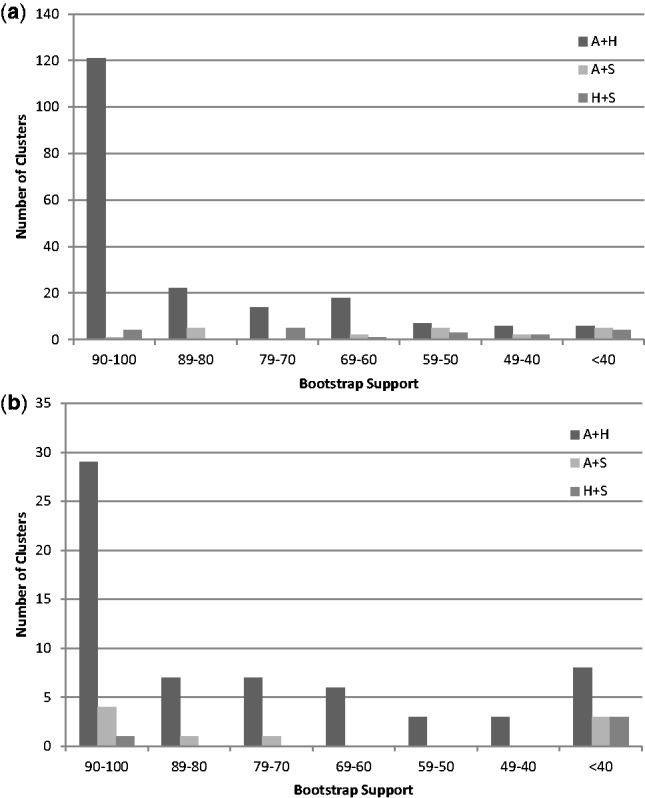
Bootstrap distributions for the pairings of different Aquificae among (*a*) 233 cohesive and (*b*) 76 noncohesive maximum likelihood trees of the AHS core subset where A, *Aquifex*; H, *Hydrogenobaculum*; *S*, *Sulfurihydrogenibium*.

To assess the cohesion of the three sampled members of the Aquificae phylum, we focused on the 527 phylogenetic profiles containing single-copy representatives of all three Aquificae genomes (AHS), examining presence and absence patterns for the Archaea (R), Epsilonproteobacteria (E), and Thermotogae (T). Analysis of the phyletic distribution (fig. 3*a*) of the AHS clusters revealed that only a small proportion of profiles were restricted to Aquificae (AHS-ø; 8; 2%). A larger number of profiles were E-only (E-ø; 38; 7%) than T-only (T-ø; 18; 3%). The two most-frequent phyletic patterns were derived from 350 (66%) ET-* profiles (i.e., RET and ET subsets) and were distinguished by the presence (229 RET) or absence (121 ET) of Archaea. The ranked Blast approach identified 337 (64%) AHS clean profiles consisting of 154 RET (67% of total RET count) and 76 ET (62% of total ET count) sets, and 190 (36%) dirty AHS profiles consisting of 75 RET (33% of total RET count) and 45 ET (38% of total ET count) sets. A total of 160 (71%) RET and 77 (65%) ET profiles produced trees with a homogeneous (cohesive) Aquificae clan (fig. 4*a*); trees of the remaining 65 (29%) RET and 42 (35%) ET profiles yielded a heterogeneous (noncohesive) Aquificae (fig. 4*b*). Situations where clean profiles yielded noncohesive clans or dirty profiles yielded cohesive clans were observed more frequently in ET (30%) than in RET (18%) sets. In such cases, we consider the phylogenetic trees to be more reliable, because they represent an explicit evolutionary model that considers all sequences at once, rather than just the weighted dissimilarity (i.e., Blast score) between pairs of sequences. Evidence from phylogenetic profiles and trees suggests that the Aquificae do constitute a distinct lineage, albeit one that is frequently affected by LGT from other sources. Disruption of Aquificae in a tree may result either from introgression of a gene from another lineage, or donation of an Aquificae gene to another lineage.

Of the 350 ET-* profiles identified earlier, six clusters contained large sequence sets (>1,500 sequences) generated by multiple non-Aquificae copies, which were not subjected to phylogenetic analysis. Two hundred of the remaining 344 (58%) trees had an associated bootstrap support value of 70% or greater for the pairing of *Aquifex* + *Hydrogenobaculum*, as compared with only 4% (12/344) supporting *Aquifex* + *Sulfurihydrogenibium* and 3% (10/344) supporting *Hydrogenobaculum* + *Sulfurihydrogenibium*. A further 10% (35/344) contained no grouping of the Aquificae (fig. 4*a* and *b*). The dominant branching pattern of *Aquifex* + *Hydrogenobaculum* as an adjacent group together with *Sulfurihydrogenibium* in a cohesive clan, is consistent with 16S ribosomal RNA gene-based taxonomy (Cole et al. 2009), which places *Aquifex* and *Hydrogenobaculum* together in family Aquificaceae, with *Sulfurihydrogenibium* joining the other two only at the class level (Aquificales).

### Affinities of Aquificae with Other Groups

To assess the overall genomic affiliations of the three Aquificae, phylogenetic profiling analysis was performed across all sets in which one or more Aquificae genome was represented (fig. 3*a*), with a focus on the counts of profiles that contained different combinations of the groups Thermotogae, Epsilonproteobacteria, and Archaea. Of the 2,019 single-copy profiles identified earlier, 779 (39%) were found only in the Aquificae. Of the remaining 1,240 clusters, 778 (63%) were present in at least one epsilonproteobacterial genome (E-*), 700 (56%) in at least one archaeal genome (R-*), and 578 (47%) in at least one member of Thermotogae (T-*). Similar trends were identified among the exclusively shared profiles, with 153 (13%) clusters exclusive to Epsilonproteobacteria (E-ø), 143 (12%) to Archaea (R-ø), and 37 (7%) to Thermotogae (T-ø). Within the E-* subset of profiles, more matches were found to the thermal vent Epsilonproteobacteria *Nitratiruptor* sp. SB155-2 (588; 76%) and *Sulfurovum* sp. NBC37-1 (550; 71%) than to any other single epsilonproteobacterium. In the subset of 527 profiles that covered all Aquificae (AHS), the E-* count (432; 83%) was greater than T-* (387; 75%) and R-* (315; 61%) suggesting that the core AHS clusters are less likely to be influenced by LGT from the Archaea than the variable clusters (fig. 3*a*).

The most notable Aquificae connections were to organisms such as the Deltaproteobacteria from the genus *Geobacter* (416–424 depending on *Geobacter* species and strain; 80–82%) and the thermophilic member of the Nitrospirae *Thermodesulfovibrio yellowstonii* DSM 11347 (420; 81%): each of these had more matches than did the thermal-vent Epsilonproteobacteria (389; 75%). Other frequently observed matching genomes were the hyperthermophilic members of the Clostridia *Carboxydothermus hydrogenoformans* Z-2901 (381; 73%) and *Thermoanaerobacter tengcongensis* MB4 (352; 68%) and the thermophilic member of the Clostridia *Caldicellulosiruptor saccharolyticus* DSM 8903 (357; 69%). Interestingly, the phylogenies of the 160 core RET clusters independently identified more trees in which one or more *Geobacter* (120; 75%) or Clostridia (116; 73%) were adjacent to a homogeneous (cohesive) Aquificae clan than was the case for Epsilonproteobacteria, Thermotogae, or Archaea.

The Deltaproteobacteria and Clostridia have been shown to contain a vast repertoire of genes believed to have been acquired by LGT (Beiko 2011; Gophna et al. 2006; Dagan et al. 2010) from other organisms including the Aquificae; the majority of these relationships cannot reflect vertical signal (especially those involving the Gram-positive Clostridia), and the substantial number of affinities with many different groups raises the question of whether Aquificae can be phylogenetically placed at all without giving special status to a small subset of genes such as those encoding the ribosome (including the 16S rRNA gene).

The analysis of Wu et al. (2009) demonstrates that different members of phylum Aquificae show varying degrees of affinity for other lineages. To assess the affiliations of each Aquificae genome individually, comparisons between the phyletic patterns of the lineage-restricted subsets (e.g., Aquifex-only: A) and the inclusive (*) Aquificae subsets (e.g., Aquifex and possibly others: A, AH, AS, and AHS) were performed (fig. 3*b*). Each inclusive Aquificae subset (colored bars in fig. 3*b*) showed a similar breakdown of affinities to other major lineages: affinities with Epsilonproteobacteria were observed more frequently than Archaea (R-*), which were in turn more frequent than Thermotogae. Among the Aquificae-only sets (gray bars in fig. 3*b*), 704 (54%) were represented in only a single member of the Aquificae (i.e., “orphan” proteins with respect to the set of 774 genomes considered), with H highest (37%), followed by S (36%) and A (28%). Comparisons of the remaining 500 lineage-specific profiles against the three inclusive Aquificae subsets (A-*, H-*, and S-*) identified the *Aquifex* genome to be strongly influenced by both the Archaea (R-*: 99; 56%) and Thermotogae (T-*: 50; 28%), *Hydrogenobaculum* by only the Archaea (R-*: 60; 57%) and *Sulfurihydrogenibium* by only Epsilonproteobacteria (E-*: 112; 51%).

### System-Level Analysis

Following the bulk characterization of taxonomic affinities of Aquificae proteins, we assessed the contributions of Archaea, Epsilonproteobacteria, and Thermotogae affiliations in terms of their functional role in the cell. If most or all constituents of a molecular system show similar patterns of inheritance, subsystem analyses may tell us more about the global Aquificae affinities and the potential origins of the Aquificae than aggregated counts of individual gene phylogenies (Doolittle and Zhaxybayeva 2009).

All 2,295 single and multi-copy clusters were classified according to the clusters of orthologous groups (COG; table 2) and Kyoto Encyclopedia of Genes and Genomes (KEGG) pathways databases to identify broad functional groups and metabolic/regulatory pathways with interesting phyletic patterns (tables 2 and 3). 63% (1,541/2,433) of all clusters were labeled with one or more COG designations and mapped to one of four broad categories with 36% (552/1,541) labeled as metabolism, 24% (368/1,541) as cellular processing and signaling, 19% (296/1,541) as information storage and processing and the remaining 21% (325/1,541) were poorly characterized.

**Table 2:** Phyletic Pattern Breakdown of All 2,433 Aquificae COGs at the Parent Level Classification across All Aquificae Subsets

Parental Category	RET	ET	RE	RT	R	E	T	Aq-Only	Other	Total	R-*	E-*	T-*
Cellular processes and signaling	94	69	33	11	18	42	8	48	45	368	156	238	182
Information storage and processing	104	66	16	18	18	10	12	34	18	296	156	196	200
Metabolism	253	25	103	31	49	39	3	23	26	552	436	420	312
Poorly characterized	62	20	59	24	77	88	21	708	158	1,217	222	229	127

**Table 3:** Phyletic Pattern Breakdown of Four Biological Subsystems of Interest Identifying the Number of Ubiquitous, Inclusive and Exclusive R, E, and T Profiles

KEGG Pathways	RET	R-*	E-*	T-*	E Not T	T Not E	R Not E	R Not T
Ribosome	17	18	40	43	2	4	1	0
Flagellar assembly	0	0	16	17	1	2	0	0
Lipopolysaccharide biosynthesis	1	3	10	1	9	0	0	2
Oxidative phosphorylation	13	29	29	16	14	0	8	15

**Fig. 5.—:**
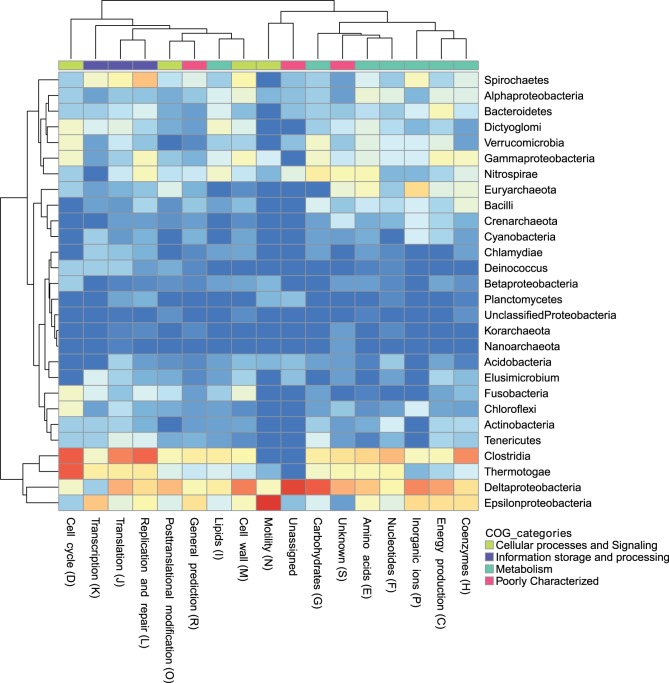
Heatmap showing the affinities of Aquificae proteins for other taxonomic groups according to a set of 315 phylogenetic trees. Each cell shows the relative proportion of trees of a given functional category that pair Aquificae with another group with an associated bootstrap value of at least 70%. The color gradient is from dark blue (very few trees supporting a given relationship) to red (a strong majority of all trees supporting that relationship).

Figure 5 shows a summary of phylogenetic affinities between Aquificae and other phyla, with Firmicutes and Proteobacteria divided into their constituent classes. Trees in which an Aquificae + X grouping (with X constituting one or more major lineages) with minimum bootstrap support of 70% was observed were interpreted as a strong pairing of Aquificae with all members of X. Substantial differences in affinity for different lineages are evident, with Deltaproteobacteria, Epsilonproteobacteria, Clostridia, and Thermotogae showing strong connections in many functional categories. As noted earlier, in many cases the Clostridia and Deltaproteobacteria show stronger affinities with Aquificae than any other group; however, as indicated earlier, these two classes show equally strong connections to many different lineages in addition to the Aquificae, likely due to their phylogenetic and functional diversity. Cell cycle and informational classes provide the strongest links with Thermotogae, whereas transcription and motility are prominent in linking Aquificae with Epsilonproteobacteria. Weaker connections with other groups such as Spirochaetes, Gammaproteobacteria, and Euryarchaeota were also observed for different subsets of functional categories.

**Fig. 6.—:**
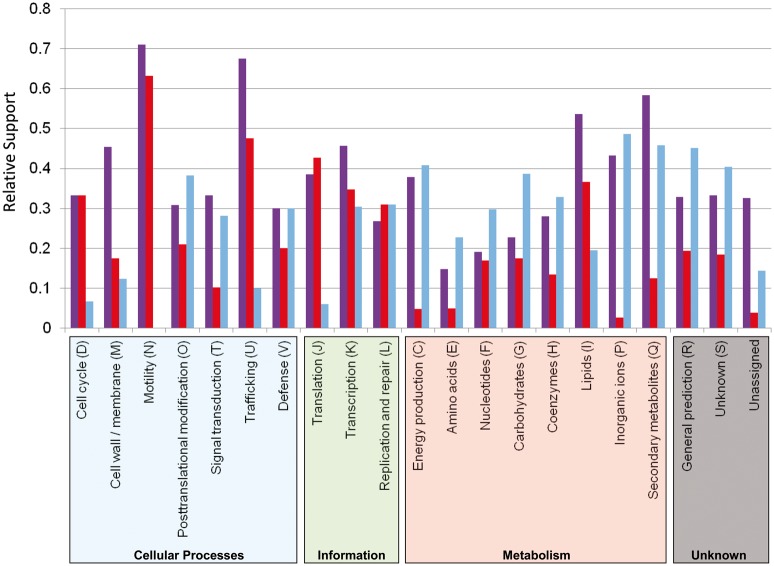
Relative support for the affinities of Aquificae with Archaea (blue), Epsilonproteobacteria (purple), Thermotogae (red) evaluated with the VPI, which expresses the number of nonubiquitous profiles that contain inclusive Archaea, Epsilonproteobacteria, or Thermotogae counts relative to the total number of profiles, excluding those found only in the Aquificae.

To assess the relative degree of support for affinities of Aquificae with R, E, and T, we computed a VPI that expresses the number of nonubiquitous profiles that contained R, E, or T relative to the total number of profiles, excluding those found only in the Aquificae. Comparison of the VPI values for each of the three distinct lineages across the 21 COG categories (supplementary table S1, Supplementary Material online) indicates the degree to which different lineages were found in association with the phylum Aquificae. As partner to Aquificae, the epsilonproteobacterial group was dominant in a handful of categories (fig. 6), notably cell wall biogenesis (M), intracellular trafficking and secretion (U), and lipid biosynthesis and transport (I); and to a lesser extent transcription (K) and secondary metabolites (Q). The Archaea were frequent partners in many metabolic categories, with the notable exception of lipids which differ substantially in their composition between Bacteria and Archaea, posttranslational modification and proteins of unknown function. Translation (J) was the only one of 21 COG categories in which Thermotogae have the largest VPI score; even in this case their score (0.43) was not considerably greater than that of the Epsilonproteobacteria (0.38). The Epsilonproteobacteria were well represented in all 21 categories, with a VPI score that was always greater than half of the best VPI score for a given category. Conversely, in many cases the VPI for either Thermotogae or Archaea was much lower than that of the two other lineages. The Archaea had VPI scores less than 0.15 for several cellular process categories including cell cycle, cell wall biosynthesis, motility (VPI = 0), and trafficking; outside this group, translation and unassigned functions also had VPI scores less than 0.15. The Thermotogae had low VPI scores in the signal transduction category, along with energy production, and the metabolism of amino acids, coenzymes, inorganic ions, and secondary metabolites. Cases in which VPI for Epsilonproteobacteria was lowest (<0.2) correspond to functions that are most widespread: Over half of all protein sets in the amino acid and nucleotide transport categories have RET distributions, which uniformly decrease all VPI scores because ubiquitous profiles can only decrease the VPI.

Deeper analysis of the profiles showed the sensitivity of the VPI measure to taxonomic sampling, particularly regarding the much broader set of Archaea (26 genera) relative to Thermotogae (4 genera) or Epsilonproteobacteria (7 genera). Even in those cases where Archaea had the highest VPI score in a particular functional category, the relative abundance of archaeal genera was relatively low. For example, profiles from the Amino Acids group (E) contained approximately 56% of archaeal genera on average, as compared with 52% of genera from Thermotogae and 72% of genera from the Epsilonproteobacteria. The high VPI associated with Archaea in some cases appears to be a consequence of lineage-specific LGT with Aquificae, whereas the affinities with Thermotogae and Epsilonproteobacteria are observed more consistently across their entire diversity of genera.

The primary focus and debate regarding the descent of the Aquificae revolves around the identification of genetic markers that appropriately describe Aquificae’s relationships among the Epsilonproteobacteria, Thermotogae and Archaea. Analysis of phylogenetic profiling coupled with functional classification identified translation (J), cell wall biosynthesis (M), and cell motility (N) to each contain distinct affiliations that may help differentiate between the two opposing Aquificae hypotheses (fig. 1). Moreover, supertree analyses revealed that phylogenies of genes involved in protein synthesis and cell wall biosynthesis (specifically the “biosynthesis of murein sacculus and peptidoglycan” role category) were less discordant when compared against the “species” tree than the protein-coding genes of cell motility (Beiko et al. 2005). These results conflict with the a priori assumptions of the complexity hypothesis (Jain et al. 1999) whereby genes encoding cell wall biosynthesis proteins were thought to be more susceptible to LGT. Other studies (Plötz et al. 2000; Cavalier-Smith 2002; Slonim et al. 2006; Wang et al. 2010) suggested that particular components of this subsystem are highly conserved across different Gram-negative bacteria and inherited vertically. Conversely, the archaeal affiliation (Aravind et al. 1998) of energy metabolism (oxidative phosphorylation) suggests that particular genes or complexes involved in respiration were acquired from the Archaea to confer some niche-specific adaptation (Boucher et al. 2003).

### Information Storage and Processing: Ribosome Structure and Biogenesis

Ribosomal proteins are widely distributed informational genes containing many protein–protein and protein–rRNA interactions thought to be refractory to transfer between divergent species (complexity hypothesis; Jain et al. 1999). Thus, the ribosomal proteins are frequently used in microbial classification to infer organismal phylogeny through the use of concatenated phylogenies (Matte-Tailliez et al. 2002; Ciccarelli et al. 2006). However, the barriers to transfer of ribosomal components are not absolute: for instance, Asai et al. (1999) showed that in *Escherichia coli* the rRNA operon can be successfully replaced by that of a distantly related species; furthermore, cases of LGT have been observed in the rps14 ribosomal protein in Bacteria (Brochier et al. 2000). To further understand the evolutionary processes and LGT susceptibility of the Aquificae translational machinery, 35 ribosomal proteins consisting of 20 large subunit (LSU) and 15 small subunit (SSU) proteins were selected from a reduced set of 47 KEGG-annotated (ko03010) clusters by removing 6 Aquificae-restricted clusters (L29, L30, L32, L35, S20, and S21) and an additional seven clusters determined by Brochier et al. (2005) to contain evidence of LGT within the archaeal domain. The reduced data set contained a broad distribution of phyletic signatures (RET, RT, ET, T, E, and Other) with similar Thermotogae (T-*; 31; 91%) and Epsilonproteobacteria (E-*; 30; 88%) profile counts (VPI: T: 47% and E: 41% and R: 6%) and phylogenetic analysis identified 23 of 34 trees (67%) where the Thermotogae were adjacent to a cohesive Aquificae clan; however, in all of these trees the sister to Aquificae consisted of Thermotogae coupled with at least one other major lineage such as Clostridia, Deltaproteobacteria, or Epsilonproteobacteria.

**Fig. 7.—:**
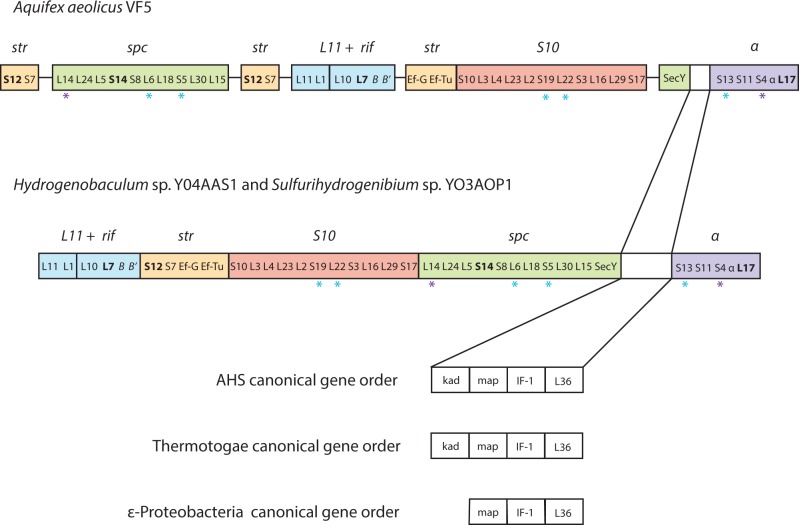
Linear arrangement of five ribosomal operons: *L11 + rif* (blue), *str* (orange), *S10* (red), *spc* (green), and *α* (purple) and their respective gene order in *Aquifex*, *Hydrogenobaculum*, and *Sulfurihydrogenibium*. Purple asterisks represent Aquificae genes with epsilonproteobacterial affinity and blue asterisks indicate archaeal affinities. *Aquifex* contains a novel operon arrangement where the majority of operon are located in different genome locations (denoted by a line separating the operons), whereas *Hydrogenobaculum* and *Sulfurihydrogenibium* have a compact operon arrangement with four genes (adenylate kinase [kad], methionine aminopepitase [map], translation initiation factor 1 [IF-1], and ribosomal L36) separating the *spc* and *α* operons. The gene order was conserved among all Aquificae and Thermotogae; however, among the Epsilonproteobacteria, kad gene is absent.

Studies of the *E. coli* ribosome identified six large operons that contain about one-half of the protein-coding genes: *str*, *spc*, *S10*, *α*, and *L11* + *rif*, with the remaining genes scattered around the genome in clusters of size one to four (Lindahl and Zengel 1986). Among the three Aquificae, the order of the five operons in *Hydrogenobaculum* and *Sulfurihydrogenibium* genomes is *L11* + *rif* + *str* + *S10 + spc + X + α*, where X comprises adenylate kinase (*kad*), methionine aminopeptidase (map), translation initiation factor 1 (*IF-1*), and ribosomal protein L36 (fig. 7).The arrangement in *Aquifex* is *S10* + *X* + *α* + *str + spc + str + L11* + *rif*, and differences with respect to the other two Aquificae cannot be explained with a single rearrangement event. Interestingly, the same ordering of the four genes in *X* is found in the Thermotogae and Dictyoglomi lineages but not among the Epsilonproteobacteria, which lack the kad gene. Bipartition analysis of the 25 coregulated genes present in the five operons identified the Thermotogae as frequently (72%; 18/25) adjacent to the Aquificae clan, although the proximity of these two groups to the exclusion of Epsilonproteobacteria and Archaea was not always strongly supported by the bootstrap analysis. Among the remaining coregulated genes, the adjacent S19 and L22 genes in the *S10* operon, the *L6* and *S5* genes of the *spc* operon and *S13* of the *α* operon were adjacent to Euryarchaeota, and *L14* and *S4* of the *spc* and *α* operons, respectively, were adjacent to the Epsilonproteobacteria. Two scenarios best explain the consistent Thermotogae affiliation observed among the single-gene phylogenies. Either 1) the Aquificae have stronger affinities with the Thermotogae (Boussau, Guéguen et al. 2008) and the archaeal (e.g., L22; Coenye and Vandamme 2005) and epsilonproteobacterial affiliations represent single-gene acquisitions or, conversely, 2) the six operons were acquired laterally from the Thermotogae followed by subsequent transfers from the archaea and Epsilonproteobacteria.

Genes encoding the nine ribosomal proteins (*L13*, *L19*, *L20*, *L21*, *L31*, *S2*, *S6*, *S9*, and *S15*), which were scattered around the Aquificae genomes in small clusters, lacked matches to the Archaea and contained identical inclusive Thermotogae and Epsilonproteobacteria profile counts (E-* and T-*: 7/9; 78%). Bipartition analysis of the six ribosomal proteins containing ET profiles revealed the same trend, identifying three trees where either Thermotogae or Epsilonproteobacteria were more often adjacent to the Aquificae than the other. Additionally, gene order conservation among the three Aquificae differed; *Sulfurihydrogenibium* and frequently *Hydrogenobaculum* contained similar gene arrangements as were found in Epsilonproteobacteria.

### Cell Motility: Flagellar Assembly

The bacterial flagellar system is both a motor organelle and a protein export/assembly apparatus extending from the cytoplasm to the cell exterior which plays a central role in cell motility, adhesion, biofilm formation, and host invasion (Harshey 2003). Recent evolutionary analysis of the flagellar complex (Liu and Ochman 2007) suggests that the core flagellar genes were derived from a single ancestor through successive duplications and diversifications where LGT played only a minor role. However, Doolittle and Zhaxybayeva (2007) refuted this claim arguing that faulty Blast settings, the disregard of seven discordant gene trees (potential LGT-driven events) in a 14-gene concatenated phylogeny, and biased comparisons of the concatenated tree with the “species” tree (reconstructed mostly from ribosomal proteins) underestimated the role of LGT. To further investigate evolutionary processes and LGT susceptibility of the Aquificae flagellar assembly, 23 Aquificae clusters were identified as components of the flagellar assembly pathway (ko02040). Removal of seven Aquificae-restricted (*flgA*, *flgB*, *flgD*, *flgL*, *flgM*, *fliE*, and *fliN*/*fliY*) and three multicopy clusters (*flhB*, *motB*/*motB*-like, and FlgG + FlgE) reduced the data set to 13 putatively orthologous clusters. The observed phyletic patterns were consistent with the nonhomologous origins of the bacterial and archaeal flagellar machinery (Ng et al. 2006) with all profiles lacking archaeal (R) signal. Phylogenetic analysis identified nine trees in which the thermophilic epsilonproteobacterium *Nitratiruptor* sp. SB155-2 was sister to *Sulfurihydrogenibium* within the Aquificae clan. Bipartition analysis revealed that seven trees (54%; 7/13) contained the mesophilic Epsilonproteobacteria (Liu and Ochman 2007) adjacent to the Aquificae + *Nitratiruptor* group while in the remainder of cases (46%; 6/13) this group was adjacent to a Thermotogae clan.

Gene ordering in the flagellar operons of the Aquificae was similar to the well-studied regulon of *Salmonella enterica* serovar *typhimurium* (Chilcott and Hughes 2000) and revealed that the ordering of 30 flagellar genes was well conserved between the thermophilic *Nitratiruptor* sp. SB155-2 and *Sulfurihydrogenibium*, suggesting recent LGT of the large flagellar regulon from *Sulfurihydrogenibium* to *Nitratiruptor*. This is consistent with the identification of a large genomic region among *Nitratiruptor* sp. SB155-2 exhibiting an atypical G + C content. The flagellar genes were also shown to exhibit the highest degree of similarity to Aquificae (i.e., *A. aeolicus*; Nakagawa et al. 2007).

### Cell Wall Biosynthesis: Lipopolysaccharide and Peptidoglycan

The bacterial cell wall is composed of peptidoglycan and, in the case of Gram-negative bacteria, a second lipid membrane containing lipopolysaccharides and lipoprotein surrounding the thin layer of peptidoglycan. Previous ultrastructure (Plötz et al. 2000; Cavalier-Smith 2002) and phylogenetic studies (Beiko et al. 2005) have suggested that cell wall proteins, most notably the peptidoglycans, tend to be inherited vertically and informative for classification, whereas Boussau, Guéguen et al. (2008) claimed that these operational genes were likely to be of significant adaptive value and suggested that the resemblance of the outer membrane between *Aquifex* and other Proteobacteria was a result of LGT.

A prominent constituent of the outer leaflet of the outer membrane in Gram-negative bacteria is lipopolysaccharide (LPS), which is composed of O-antigen repeats, core oligosaccharide region and the membrane-anchoring lipid A molecule (Raetz et al. 2007). Extensive studies of the lipopolysaccharide biosynthesis (ko00540) pathways in *E. coli* and other bacteria has revealed that the structure of lipid A as well as the enzymes involved in biosynthesis of the molecule were more widely conserved in different Gram-negative bacteria (Slonim et al. 2006) than the core oligosaccharide or O-antigen repeats (Wang et al. 2010). Amongst all three Aquificae, only lipid A and 3-deoxy-d-manno-oct-2-ulosonic acid (Kdo) synthesis were inferred to be present, whereas the presence of the O-antigen enzymes was variable.

The first stage in the biosynthesis of the LPS is the synthesis of the Kdo_2_-lipid A. Eight of the nine Aquificae clusters implicated in Kdo_2_-lipid A synthesis were shared exclusively with the Epsilonproteobacteria, while the Thermotogae and Archaea were represented only in one profile (KdsA, which is present in many Gram-positive organisms: Slonim et al. 2006). Two additional enzymes in the pathway, LpxL and LpxM, were not detected in *Aquifex* and *Hydrogenobaculum*; LpxL was present in *Sulfurihydrogenibium* and other mesophilic and thermophilic Epsilonproteobacteria. Similarly, the KdsA, KdsC, and KpsU enzymes involved in Kdo synthesis produced trees with mesophilic and thermophilic Epsilonproteobacteria branching with a cohesive Aquificae clan.

Peptidoglycan (PG) is a major component of the cell wall of most prokaryotes and helps to maintain cell shape and provide mechanical strength to resist osmotic pressures (Typas et al. 2011). Although the PG enzymes themselves do not associate into large multisubunit complexes, the molecules they synthesize must be cohesive and able to interact with the existing PG structures of the cell (Vollmer and Bertsche 2008). Consequently, transfer of PG synthesis genes may carry a substantial selective cost. The biosynthesis of peptidoglycan involves numerous cytoplasmic steps to synthesize two lipid intermediates, Lipid I and Lipid II, and to transport the latter across the bacterial membrane. In the three Aquificae, 14 protein-coding genes associated with peptidoglycan biosynthesis (ko00550) pathway were identified. Phylogenetic and bipartition analyses of the enzymes involved in cytoplasmic steps (MurA, MurB, MurCDEF, Ddl, and MurI) identified an Aquificae cohesive clan adjacent to the Epsilonproteobacteria in the trees of MurA, B, and I, whereas the Mur ligases MurCD and MurEF, and Ddl were adjacent to the Thermotogae. The enzymes involved in the biosynthesis of the peptidoglycan lipid-linked intermediates, MraY and MurG, the flippase FtsW-RodA, and the enzymes involved in the polymerization reactions, mrcA/PBP1A, mrdA/PBP2, and FtsI/PBP3 placed the Aquificae with the Epsilonproteobacteria.

### Energy Metabolism: Oxidative Phosphorylation

The oxidative phosphorylation process (ko00190) forms ATP as a result of the transfer of electrons from NAHD or FADH_2_ to a final electron acceptor (usually molecular oxygen; O_2_) through a series of electron carriers. The flow of electrons through a sequential set of large proton-pump supercomplexes, nicotinamide adenine dinucleotide (NADH) dehydrogenase (complex I), cytochrome *c* reductase (complex III), and cytochrome *c* oxidase (complex IV) generates an electrochemical potential gradient that drives the production of ATP by *F_0_F_1_* ATP synthase (complex V). Evidence has been accumulating over the past few decades that respiratory chains are dynamic systems that display great variability in their components. Studies have revealed that the genes involved in these essential complexes have experienced frequent exchange across vast phylogenetic distances (Hilario and Gogarten 1993). However, given the number of genes involved in this process, strong affinities between Aquificae and a single group across all five complexes would support either vertical inheritance of most genetic components of this system, or extreme convergence of these groups due to many independent LGT events. Absence of a single, strong signal would support piecemeal assembly through LGT.

The respiratory chain of the Aquificae contained 37 clusters: 13 in complex I, 2 in complex II (succinate dehydrogenase/fumarate reductase), 3 in complex III, 10 in complex IV, and 9 in complex V. Phylogenetic profiling of the five complexes identified similar affinities of the Aquificae to Archaea (24 R-*) and Epsilonproteobacteria (22 E-*), and fewer genes in common with the Thermotogae (10 T-*). The anaerobic Thermotogae lack the majority of aerobic complexes with the exception of nine genes from complex I and all of the ATP synthase proteins (Slonim et al. 2006).

Complex I or NADH dehydrogenase is the first entry point for electrons into the respiratory electron transport chain. In bacteria, the structure (Clason et al*.* 2010) is comprised of 14 core Nuo proteins (Hirst 2010), two of which (C and D) are fused in a range of organisms including *E. coli* and *A. aeolicus* (Scheide et al. 2002). The complex can be subdivided into soluble (E, F, and G), amphipathic (B, CD, and I), and hydrophobic, membrane-bound (A, H, J, K, L, M, and N) components (Leif et al. 1995). The Aquificae were cohesive in trees of these proteins, with the exception of the *Aquifex* NuoL2, which branched with the Alphaproteobacteria. When relationships to the Thermotogae, Epsilonproteobacteria, and Archaea were considered, the Aquificae were adjacent to the Epsilonproteobacteria in trees of all hydrophobic components and the amphipathic components CD and I, and nearer to the Euryarchaeota in the case of amphipathic subunit B and the soluble subunits E, F, and G.

Complex II transfers electrons from FADH_2_ to Fe–S centers by succinate dehydrogenase (Sdh), ultimately reducing ubiquinone (Q) to ubiquinol (QH_2_). Both *Aquifex* and *Hydrogenobaculum* contained catalytic Sdh and cluster-containing FrdB genes, while *Sulfurihydrogenibium* also contained the cluster-containing *sdhA* gene. Phylogenetic and bipartition analysis placed the mesophilic Epsilonproteobacteria adjacent to a cohesive Aquificae in SdhA phylogeny and the SdhB/FrdB phylogeny placed the five Aquificae FrdB copies adjacent to the euryarchaeotes, whereas the *Sulfurihydrogenibium* SdhB protein sequence branched with mesophilic Epsilonproteobacteria. These observations suggest that SdhB was functionally replaced in *Aquifex* and *Hydrogenobaculum* by an archaeal FrdB homolog and the ancestral SdhB homolog was simultaneously or subsequently lost. The three proteins of Complex III showed a similar pattern, with Aquificae adjacent to the Epsilonproteobacteria for two proteins (ISP and Cyt1), whereas in the Cytb tree the Aquificae were not cohesive, with *Sulfurihydrogenibium* adjacent to the Epsilonproteobacteria while the other two Aquificae branched with a group that includes Archaea, Deltaproteobacteria, and Actinobacteria.

Complex IV catalyzes the transfer of electrons from reduced cytochrome *c* (cytochrome *c* oxidase) or quinol (quinol oxidase) to the final acceptor (usually molecular oxygen), ultimately translocating four protons. The three Aquificae contain three different oxidases, with the *aa_3_*-type present only in *Aquifex*, the minimal *cbb_3_*-type cytochrome *c* oxidase found in both *Hydrogenobaculum* and *Sulfurihydrogenibium*, and *bd*-type quinol oxidase present in all three Aquificae. Gene order of the *Aquifex aa_3_*-type oxidase, unlike the subunit COXII-I-III-IV ordering observed in *E. coli* and *Bacillus subtilis* (García-Horsman et al. 1994), contained two adjacent operons separated by a 550 bp intergenic spacer. Phylogenies of the subunits COXII, I of the second operon identified *Aquifex* adjacent to the Archaea whereas COXIII, II, and I of the first operon were adjacent to a proteobacterial clan, which did not include any Epsilonproteobacteria. Among the heme biosynthesis genes, the heme O-generating CyoE/CtaB preferentially branched with the Proteobacteria (*α*, *β*, and *γ*), whereas the COX15/CtaA-heme A gene branched with the Archaea. The *cbb_3_*-type found in *Hydrogenobaculum* and *Sulfurihydrogenibium* both contain a single subunit, COX1, that was adjacent to the Epsilonproteobacteria, other Proteobacteria and Clostridia. The three Aquificae utilize another high oxygen affinity complex IV—*bd*-type quinol oxidase that is not a member of the heme–copper superfamily and contains a modified heme B–heme D in subunit COXII. This enzyme complex is a membrane-bound heterodimer encoded by two subunits CydA and CydB, which were both found to branch with the Archaea.

Complex V, ATP synthase, is the final enzyme supercomplex in the oxidative phosphorylation pathway, which synthesizes ATP from ADP and inorganic phosphate (Pi) generated by the downhill flow of protons, produced by complexes I, III, and IV, across the inner membrane. The highly conserved and ubiquitous F_0_F_1_ ATP synthase is composed of five hydrophilic components of the F_1_ complex (*α*, *β*, *δ*, *ε*, and *γ* subunits) which catalyzes ATP hydrolysis/synthesis and the three transmembrane-containing a, b, and c subunits of the F_0_ complex which acts as the proton channel (Yoshida et al. 2001). Phylogenies and bipartition analysis of the F_0_ complex placed the Aquificae with the mesophilic Epsilonproteobacteria, whereas the second copy of subunit b was adjacent to a heterogeneous Epsilonproteobacteria + Thermotogae clan. The F_1_ components showed weak bootstrap support for the grouping of the Aquificae with the Epsilonproteobacteria with the exception of the noncatalytic α subunit, which was clearly Epsilonproteobacteria derived.

### Subsystem Phylogenetic Cohesion

Each of the subsystems outlined earlier was subjected to a concatenated analysis. In each case, a single phylogenetic tree was constructed from the concatenated alignment of all protein sets with sufficient taxonomic coverage. In most cases, trees built from concatenated alignments showed similar relationships for the Aquificae as were seen in the majority of the individual protein trees (table 4). Trees built from concatenations of ribosomal proteins showed a classic early-branching position for the Aquificae, although large and small-subunit trees differed in the branching order of Aquificae, Thermotogae, Deinococcus-Thermus, and Dictyoglomi. Trees built from other subsystems tended to group Aquificae with Epsilonproteobacteria, albeit often with other groups such as Euryarchaeota as sisters as well.

**Table 4:** Concatenated Analysis of Proteins from Selected Functional Subsystems

System	Subsystem	Number of Trees in Concatenation	Closest Neighboring Phyla or Classes	Minimum *P*-Value	Maximum *P*-Value	Number Rejected
Cell wall	LipidA	9	Epsilonproteobacteria, Fusobacteria, Bacilli	6e–125	3e–05	7

	Peptidoglycan	15	Epsilonproteobacteria	2e–133	2e–04	14

Flagella		13	Verrucomicrobia, Gammaproteobacteria, Epsilonproteobacteria	5e–89	4e–04	13

Oxidative phosphorylation	CI	12	Euryarchaeota, Korarchaeota, Epsilonproteobacteria	2e–132	5e–01	11

	CII	2	Chlorobi, Deltaproteobacteria	4e–42	5e–30	2

	CIII	3	Several other phyla	2e–116	2e–01	2

	CIV-aa3	6	Several other phyla	2e–95	2e–02	5

	CIV-bd	2	Clostridia (*Carboxydothermus hydrogenoformans*)	3e–06	2e–04	2

	CIV-cbb3	1	N/A			

	CV	8	Euryarchaeota, Epsilonproteobacteria	9e–62	2e–02	7

Ribosome	LSU	20	Sister to all other Bacteria	3e–133	5e–02	19

	SSU	15	Sister to all other bacteria except Thermotogae, Dictyoglomi, and *Coprothermobacter proteolyticus*	5e–86	1e–04	15

Note.—For each subsystem, the total number of protein sets used in the concatenation is shown (concatenations with fewer than five proteins are not shown). The closest-matching phyla or classes are identified if three or fewer were sister to Aquificae. Extreme *P* values of AU tests carried out on each individual protein tree are shown, along with the number of trees that were rejected by the test (*P* < 0.001).

We tested the validity of our concatenated approach by statistically comparing the individual gene trees of a subsystem with trees built from the concatenated alignments. The concatenation-based tree was rarely accepted as a possible topology to describe the evolution of the individual genes (table 4). Although the specific partnerships of Aquificae may not contribute to the rejection of many gene trees, the result suggests that the observed differences among trees are due to true phylogenetic discordance and not statistical artifacts alone.

### Discussion

The Aquificae have an unusually complex evolutionary history, with a majority of their genomes potentially acquired via LGT (Aravind et al. 1998; Nelson et al. 1999). At least two plausible scenarios can be considered to describe the descent of this group that differ in the interpretation of the true extant sister of the Aquificae (fig. 1) as being either the phylum Thermotogae (Boussau, Guéguen et al. 2008) or the class Epsilonproteobacteria (Cavalier-Smith 2002; Beiko et al. 2005). *Aquifex aeolicus* VF5 was the first genome from the Aquificae phylum to be subjected to phylogenomic analysis, and its unique affiliations (fig. 3*b*) may have presented a skewed view of the genetic affinities of the phylum Aquificae. These unique affinities should be borne in mind when single representatives of deep lineages are added through technologies such as single-cell sequencing (Rinke et al. 2013).

This study includes two additional Aquificae genomes, *Hydrogenobaculum* sp. Y04AAS1 and *Sulfurihydrogenibium* sp. YO3AOP1 (Reysenbach et al. 2009), and identified a core of 527 gene sets common to all three Aquificae. Phylogenetic analyses of broadly distributed constituents of this core set of proteins revealed that the majority of these protein-coding genes exhibit identical branching patterns within the Aquificae to those seen in 16S rDNA phylogenies (Cole et al. 2009). The cohesion of this group was frequently observed in phylogenetic trees built from RET-profile proteins (i.e., phylogenetic profiles containing Blast matches to at least one member of the Archaea, Epsilonproteobacteria, and Thermotogae), suggesting that the Aquificae are indeed a distinct lineage. However, genes with ET profiles tended to produce less-cohesive trees, containing other lineages interleaved amongst the Aquificae, particularly thermophilic bacteria of the Epsilonproteobacteria (*Nitratiruptor* sp. SB155-2), and Nitrospira. Furthermore, many proteins in this core set also showed affiliations with mesophiles from the Epsilonproteobacteria and Deltaproteobacteria. These results imply that LGT is, indeed, rampant even among the core gene set.

Functional categorization of this core Aquificae set identified a subset of genes involved in translation (category J; fig. 6) to be affiliated with the Thermotogae (Nelson et al. 1999; Boussau et al. 2008). In-depth examination of an essential component involved in translation—the ribosomal protein complex—revealed a mosaic of affiliations of the genes present among the six major ribosomal operons. Additionally, gene organization studies revealed the consistent presence of four genes (*kad*, *map*, *IF-1* and *L36*, located between the *secy* gene and *α* operon) in all Aquificae and Thermotogae (and other thermophilic lineages) while the adenylate kinase (*kad*) gene was absent from this region in all epsilonproteobacterial genomes (fig. 7). The affiliation of the Aquificae with Epsilonproteobacteria, however, dominated numerous functional categories (fig. 6), with cell envelope/outer membrane biogenesis (M) as the most striking example. Further investigations identified a pathway—lipid A biosynthesis—known to be present in most Gram-negative bacteria and absent in Gram-positive bacteria (Slonim et al. 2006). Phylogenetic analyses of the constituent enzymes from this widely conserved pathway (Mamat et al. 2009; Wang and Quinn 2010) were consistent with exclusive epsilonproteobacterial affiliations identified in previous studies (Plötz et al. 2000; Cavalier-Smith 2002; Beiko et al. 2005). Thus, the lipid A biosynthetic pathway may have been preferentially inherited among the three Aquificae for the expression of the Gram-negative trait.

If one considers our results under the assumptions of the complexity hypothesis (Jain et al. 1999) and in the context of the two scenarios depicted in figure 1, our findings suggest that the Aquificae are sister to the Thermotogae (Boussau, Guéguen et al. 2008), or at least that both groups are proximal in unrooted trees (suggesting an early branching scenario). This supposition is largely based on the analyses of a particular subset of the informational genes—the ribosomal protein complex (fig. 1*a*). The conclusion that Aquificae and Thermotogae are both early branching and ancestrally thermophilic has been supported by ribosomal RNA gene analysis (Boussau, Guéguen et al. 2008) and examination of informational genes such as EF-Tu (Gaucher et al. 2008). However, recent reevaluations of the complexity hypothesis (Hao and Golding 2008) and the numerous studies identifying LGT among informational genes (Gogarten et al. 2002; Kanhere and Vingron 2009) suggest that the functional distinction between informational and operational genes are of limited utility as a predictive tool for identifying transferred genes, and calls into question the assumption that informational genes in disparate lineages diverged long ago. Indeed, individual gene phylogenies within the six major ribosomal operons show alternative affiliations to Archaea or Epsilonproteobacteria (fig. 7), and transcriptional genes (COG class K; fig. 6) preferentially show epsilonproteobacteria*l* affiliations (Gruber and Bryant 1998; Klenk et al. 1999). Thus, another plausible interpretation of the data presented herein is that the evolution of different gene sets reflects the lifestyle of the organisms in which they reside—in this case thermophily or mesophily—rather than their functional category.

The Aquificae contain a significant fraction of genes that were potentially acquired from or donated to other thermophilic lineages, establishing a plausible connection between the similarity in lifestyle of evolutionarily distant organisms and the apparent rate of LGT (Aravind et al. 1998; Nelson et al. 1999). Indeed, many Aquificae genes, particularly those with metabolic functions, are related to the Archaea, particularly the Euryarchaeota, and were likely acquisitions enabling differing strategies for ecological adaptation, such as the *bd*-type complex IV of oxidative phosphorylation, which may confer adaptation to low oxygen concentrations. The affiliations to other thermophilic lineages, particularly the Thermotogae, *Nitratiruptor* sp. SB155-2 and Nitrospira, however, may have been acquisitions among these bacterial lineages simply due to their proximity with other thermophiles in the environment. Moreover, bacterial lineages that were initially mesophilic and later colonized a hot environment were shown to have widespread amino acid biases (i.e., a significant increase in charged residues) in their proteome (Singleton and Amelunxen 1973; Boucher et al. 2003; Berezovsky and Shakhnovich 2005). Thus, the acquisitions of essential gene sets from other thermophilic bacteria (e.g., the ribosomal complex and flagellar assembly genes) may be of selective advantage to these organisms, conferring thermal stabilization of these important protein complexes. This is in contrast to the cell membrane in which structural differences, such as the increase in saturated and branch-chained fatty acids including branched glycerol dialkyl diethers, have been proposed to contribute to the thermal stability of the membrane (Boucher et al. 2003). Thus, different types of proteins are constrained in different ways by very hot environments.

Our ability to assess the relative affinities of Aquificae for other lineages, in particular the Thermotogae, Epsilonproteobacteria, and Archaea, may be impacted by uneven taxonomic sampling of these three lineages. The three lineages differed both in the raw number of genomes available (7 Thermotogae, 22 Epsilonproteobacteria, and 53 Archaea) and the taxonomic and phylogenetic diversity (the Archaea span at least two distinct phyla, while the other two groups were much more restricted in their diversity). The impacts of sampling are attenuated somewhat by our focus on phylogenetic profiles covering Aquificae and all three other main lineages (i.e., RET profiles), and by examining closest affinities in phylogenetic trees. Inclusion of the genera *Nitratiruptor* and *Sulfurovum* demonstrates how single sampled members of particularly critical lineages can influence phylogenomic conclusions, and it is possible that future analyses containing a novel member of the Aquificae, Thermotogae or Epsilonproteobacteria will generate results that shift the balance of support in one direction or the other.

Given the patterns of phylogenetic relatedness seen in the subsystems that were investigated, we believe that the majority of genes in the Aquificae that appear related to thermophiles were likely lateral acquisitions, whereas those with epsilonproteobacterial affiliations may be remnants of a mesophilic past that predated its colonization of a thermophilic environment (fig. 1*b*). Environmental studies of hydrothermal adaptations have revealed that the Epsilonproteobacteria have developed diverse strategies to colonize many deep-sea substrates due, in part, to their high growth rates, rapid adaptations to changing geochemical conditions and metabolic versatility (Lopez-Garcia et al. 2003; Nakagawa et al. 2007). The Epsilonproteobacteria may thus be major contributors in the colonization processes where they play a vital role in the cycling of carbon, nitrogen and sulfur (Alain et al. 2004; Campbell et al. 2006). Given the high degree of mosaicism reported here, and considering the phylogenetic and physiological (e.g., cell wall biosynthesis; Cavalier-Smith 2002) evidence, it is plausible that the Aquificae are derived Epsilonproteobacteria that acquired genes that confer adaptations to a thermophilic environment. However, under such a model of extensive gene sharing, we cannot rule out the possibility that the true ancestral signal of the Aquificae may have been irrevocably lost.

## Supplementary Material

Supplementary table S1 is available at *Genome Biology and Evolution* online (http://www.gbe.oxfordjournals.org/).

Supplementary Data

## References

[evt195-B1] Alain K (2004). Early steps in microbial colonization processes at deep-sea hydrothermal vents. Environ Microbiol..

[evt195-B2] Altschul SF (1997). Gapped BLAST and PSI-BLAST: a new generation of protein database search programs. Nucleic Acids Res..

[evt195-B3] Andam CP, Williams D, Gogarten JP (2010). Biased gene transfer mimics patterns created through shared ancestry. Proc Natl Acad Sci U S A..

[evt195-B4] Asai T (1999). Construction and initial characterization of *Escherichia coli* strains with few or no intact chromosomal rRNA operons. J Bacteriol..

[evt195-B5] Aravind L, Tatusov RL, Wolf YI, Walker DR, Koonin EV (1998). Evidence for massive gene exchange between archaeal and bacterial hyperthermophiles. Trends Genet..

[evt195-B6] Baker WJ (2009). Complete generic-level phylogenetic analyses of palms (Arecaceae) with comparisons of supertree and supermatrix approaches. Syst Biol..

[evt195-B7] Baldauf SL, Palmer JD, Doolittle WF (1996). The root of the universal tree and the origin of eukaryotes based on elongation factor phylogeny. Proc Natl Acad Sci..

[evt195-B8] Bapteste E (2005). Do orthologous gene phylogenies really support tree-thinking?. BMC Evol Biol..

[evt195-B9] Beiko RG (2011). Telling the whole story in a 10,000-genome world. Biol Direct..

[evt195-B10] Beiko RG, Doolittle WF, Charlebois RL (2008). The impact of reticulate evolution on genome phylogeny. Syst Biol..

[evt195-B11] Beiko RG, Harlow TJ, Ragan MA (2005). Highways of gene sharing in prokaryotes. Proc Natl Acad Sci U S A..

[evt195-B12] Beiko RG, Keith JM, Harlow TJ, Ragan MA (2006). Searching for convergence in phylogenetic Markov chain Monte Carlo. Syst Biol..

[evt195-B13] Berezovsky IN, Shakhnovich EI (2005). Physics and evolution of thermophilic adaptation. Proc Natl Acad Sci U S A..

[evt195-B15] Bininda-Emonds OR, Sanderson MJ (2001). Assessment of the accuracy of matrix representation with parsimony analysis supertree construction. Syst Biol..

[evt195-B16] Bocchetta M (1995). Arrangement and nucleotide sequence of the gene (*fus)* encoding elongation factor G (EF-G) from the hyperthermophilic bacterium *Aquifex pyrophilus*: phylogenetic depth of hyperthermophilic bacteria inferred from analysis of the EF-G/*fus* sequences. J Mol Evol..

[evt195-B17] Bordenstein SR, Reznikoff WS (2005). Mobile DNA in obligate intracellular bacteria. Nat Rev Microbiol..

[evt195-B18] Boucher Y (2003). Lateral gene transfer and the origins of prokaryotic groups. Annu Rev Genet..

[evt195-B19] Boussau B, Blanquart S, Necsulea A, Lartillot N, Gouy M (2008). Parallel adaptations to high temperatures in the Archaean eon. Nature.

[evt195-B20] Boussau B, Guéguen L, Gouy M (2008). Accounting for horizontal gene transfers explains conflicting hypotheses regarding the position of aquificales in the phylogeny of bacteria. BMC Evol Biol..

[evt195-B21] Bradley RK (2009). Fast statistical alignment. PLoS Comput Biol..

[evt195-B22] Brochier C, Philippe H, Moreira D (2000). The evolutionary history of ribosomal protein RpS14: horizontal gene transfer at the heart of the ribosome. Trends Genet..

[evt195-B107] Brochier C, Forterre P, Gribaldo S (2005). An emerging phylogenetic core of Archaea: phylogenies of transcription and translation machineries converge following addition of new genome sequences. BMC Evol Biol..

[evt195-B24] Campbell BJ, Engel AS, Porter ML, Takai K (2006). The versatile epsilon-proteobacteria: key players in sulphidic habitats. Nat Rev Microbiol..

[evt195-B25] Cavalier-Smith T (2002). The neomuran origin of archaebacteria, the negibacterial root of the universal tree and bacterial megaclassification. Int J Syst Evol Microbiol..

[evt195-B27] Chilcott GS, Hughes KT (2000). Coupling of flagellar gene expression to flagellar assembly in *Salmonella enterica* serovar typhimurium and *Escherichia coli*. Microbiol Mol Biol Rev..

[evt195-B28] Ciccarelli FD (2006). Toward automatic reconstruction of a highly resolved tree of life. Science.

[evt195-B29] Clason T (2010). The structure of eukaryotic and prokaryotic complex I. J Struct Biol..

[evt195-B108] Coenye T, Vandamme P (2005). Organisation of the S10, spc and alpha ribosomal protein gene clusters in prokaryotic genomes. FEMS Microbiol Lett..

[evt195-B30] Cole JR (2009). The Ribosomal Database Project: improved alignments and new tools for rRNA analysis. Nucleic Acids Res..

[evt195-B32] Dagan T, Roettger M, Bryant D, Martin W (2010). Genome networks root the tree of life between prokaryotic domains. Genome Biol Evol..

[evt195-B33] Deckert G (1998). The complete genome of the hyperthermophilic bacterium *Aquifex aeolicus*. Nature.

[evt195-B34] Doolittle WF, Zhaxybayeva O (2007). Evolution: reducible complexity—the case for bacterial flagella. Curr Biol..

[evt195-B35] Doolittle WF, Zhaxybayeva O (2009). On the origin of prokaryotic species. Genome Res..

[evt195-B36] Eddy SR (2009). A new generation of homology search tools based on probabilistic inference. Genome Inform..

[evt195-B37] Felsenstein J (1989). PHYLIP—phylogeny inference package (version 3.2). Cladistics.

[evt195-B38] Gaasterland T, Ragan MA (1998). Microbial genescapes: phyletic and functional patterns of ORF distribution among prokaryotes. Microb Comp Genomics..

[evt195-B39] Galtier N (2007). A model of horizontal gene transfer and the bacterial phylogeny problem. Syst Biol..

[evt195-B40] García-Horsman JA, Barquera B, Rumbley J, Ma J, Gennis RB (1994). The superfamily of heme-copper respiratory oxidases. J Bacteriol..

[evt195-B41] Gaucher EA, Govindarajan S, Ganesh OK (2008). Palaeotemperature trend for Precambrian life inferred from resurrected proteins. Nature.

[evt195-B42] Gene Ontology Consortium (2000). Gene ontology: tool for the unification of biology. Nat Genet..

[evt195-B43] Gogarten JP, Doolittle WF, Lawrence JG (2002). Prokaryotic evolution in light of gene transfer. Mol Biol Evol..

[evt195-B44] Gophna U, Charlebois RL, Doolittle WF (2006). Ancient lateral gene transfer in the evolution of *Bdellovibrio bacteriovorus*. Trends Microbiol..

[evt195-B46] Griffiths E, Gupta RS (2004). Signature sequences in diverse proteins provide evidence for the late divergence of the Order Aquificales. Int Microbiol..

[evt195-B47] Gruber TM, Bryant DA (1998). Characterization of the group 1 and group 2 sigma factors of the green sulfur bacterium *Chlorobium tepidum* and the green non-sulfur bacterium *Chloroflexus aurantiacus*. Arch Microbiol..

[evt195-B48] Hao W, Golding GB (2008). Uncovering rate variation of lateral gene transfer during bacterial genome evolution. BMC Genomics.

[evt195-B49] Harshey RM (2003). Bacterial motility on a surface: many ways to a common goal. Annu Rev Microbiol..

[evt195-B50] Hilario E, Gogarten JP (1993). Horizontal transfer of ATPase genes—the tree of life becomes a net of life. Biosystems.

[evt195-B51] Hirst J (2010). Towards the molecular mechanism of respiratory complex I. Biochem J..

[evt195-B52] Holloway C, Beiko RG (2010). Assembling networks of microbial genomes using linear programming. BMC Evol Biol..

[evt195-B53] Huber R, Hannig M, Dworkin M (2006). Thermotogales. The Prokaryotes. An evolving electronic resource for the microbiological community.

[evt195-B54] Huber R (1992). *Aquifex pyrophilus* gen nov., sp. nov., represents a novel group of marine hyperthermophilic hydrogen-oxidizing bacteria. Syst Appl Microbiol..

[evt195-B55] Huber R (1998). *Thermocrinis ruber* gen. nov., sp. nov., a pink-filament-forming hyperthermophilic bacterium isolated from yellowstone national park. Appl Environ Microbiol..

[evt195-B56] Iyer LM, Koonin EV, Aravind L (2004). Evolution of bacterial RNA polymerase: implications for large-scale bacterial phylogeny, domain accretion, and horizontal gene transfer. Gene.

[evt195-B57] Jain R, Rivera MC, Lake JA (1999). Horizontal gene transfer among genomes: the complexity hypothesis. Proc Natl Acad Sci U S A..

[evt195-B58] Kanehisa M, Goto S (2000). KEGG: Kyoto encyclopedia of genes and genomes. Nucleic Acids Res..

[evt195-B59] Kanhere A, Vingron M (2009). Horizontal gene transfers in prokaryotes show differential preferences for metabolic and translational genes. BMC Evol Biol..

[evt195-B60] Klenk HP (1999). RNA polymerase of *Aquifex pyrophilus*: implications for the evolution of the bacterial rpoBC operon and extremely thermophilic bacteria. J Mol Evol..

[evt195-B61] Koga Y, Morii H (2007). Biosynthesis of ether-type polar lipids in archaea and evolutionary considerations. Microbiol Mol Biol Rev..

[evt195-B62] Koski LB, Golding GB (2001). The closest BLAST hit is often not the nearest neighbor. J Mol Evol..

[evt195-B63] Lapointe FJ, Lopez P, Boucher Y, Koenig J, Bapteste E (2010). Clanistics: a multi-level perspective for harvesting unrooted gene trees. Trends Microbiol..

[evt195-B64] Leif H, Sled VD, Ohnishi T, Weiss H, Friedrich T (1995). Isolation and characterization of the proton-translocating NADH: ubiquinone oxidoreductase from *Escherichia coli*. Eur J Biochem..

[evt195-B65] Leigh JW, Susko E, Baumgartner M, Roger AJ (2008). Testing congruence in phylogenomic analysis. Syst Biol..

[evt195-B66] L’Haridon S (2006). *Desulfurobacterium atlanticum* sp. nov., *Desulfurobacterium pacificum* sp. nov. and *Thermovibrio guaymasensis* sp. nov., three thermophilic members of the Desulfurobacteriaceae fam. nov., a deep branching lineage within the Bacteria. Int J Syst Evol Microbiol..

[evt195-B67] Lindahl L, Zengel JM (1986). Ribosomal genes in *Escherichia coli*. Annu Rev Genet..

[evt195-B68] Liu R, Ochman H (2007). Stepwise formation of the bacterial flagellar system. Proc Natl Acad Sci U S A..

[evt195-B69] López-García P (2003). Bacterial diversity in hydrothermal sediment and epsilonproteobacterial dominance in experimental microcolonizers at the Mid-Atlantic Ridge. Environ Microbiol..

[evt195-B70] Mamat U (2009). WaaA of the hyperthermophilic bacterium *Aquifex aeolicus* is a monofunctional 3-deoxy-d-manno-oct-2-ulosonic acid transferase involved in lipopolysaccharide biosynthesis. J Biol Chem..

[evt195-B71] Mathee K (2008). Dynamics of *Pseudomonas aeruginosa* genome evolution. Proc Natl Acad Sci U S A..

[evt195-B72] Matte-Tailliez O, Brochier C, Forterre P, Philippe H (2002). Archaeal phylogeny based on ribosomal proteins. Mol Biol Evol..

[evt195-B74] Nakagawa S (2005). Distribution, phylogenetic diversity and physiological characteristics of epsilon—proteobacteria in a deep-sea hydrothermal field. Environ Microbiol..

[evt195-B75] Nakagawa S (2007). Deep-sea vent epsilon-proteobacterial genomes provide insights into emergence of pathogens. Proc Natl Acad Sci U S A..

[evt195-B76] Nelson KE (1999). Evidence for lateral gene transfer between Archaea and bacteria from genome sequence of *Thermotoga maritima*. Nature.

[evt195-B77] Ng SY, Chaban B, Jarrell KF (2006). Archaeal flagella, bacterial flagella and type IV pili: a comparison of genes and posttranslational modifications. J Mol Microbiol Biotechnol..

[evt195-B79] Pellegrini M, Marcotte EM, Thompson MJ, Eisenberg D, Yeates TO (1999). Assigning protein functions by comparative genome analysis: protein phylogenetic profiles. Proc Natl Acad Sci U S A..

[evt195-B80] Philippe H (2004). Phylogenomics of eukaryotes: impact of missing data on large alignments. Mol Biol Evol..

[evt195-B81] Plötz BM, Lindner B, Stetter KO, Holst O (2000). Characterization of a novel lipid A containing d-galacturonic acid that replaces phosphate residues. The structure of the lipid A of the lipopolysaccharide from the hyperthermophilic bacterium *Aquifex pyrophilus*. J Biol Chem..

[evt195-B82] Puigbò P, Wolf YI, Koonin EV (2010). The tree and net components of prokaryote evolution. Genome Biol Evol..

[evt195-B83] Raetz CR, Reynolds CM, Trent MS, Bishop RE (2007). Lipid A modification systems in Gram-negative bacteria. Annu Rev Biochem..

[evt195-B84] Renesto P, Ogata H, Audic S, Claverie JM, Raoult D (2005). Some lessons from Rickettsia genomics. FEMS Microbiol Rev..

[evt195-B85] Reysenbach AL (2009). Complete and draft genome sequences of six members of the Aquificales. J Bacteriol..

[evt195-B86] Rinke C (2013). Insights into the phylogeny and coding potential of microbial dark matter. Nature.

[evt195-B88] Scheide D, Huber R, Friedrich T (2002). The proton-pumping NADH: ubiquinone oxidoreductase (complex I) of *Aquifex aeolicus*. FEBS Lett..

[evt195-B89] Schütz M (2000). Early evolution of cytochrome *bc* complexes. J Mol Biol..

[evt195-B90] Shimodaira H, Hasegawa M (2001). CONSEL: for assessing the confidence of phylogenetic tree selection. Bioinformatics.

[evt195-B91] Simmons MP (2011). Misleading results of likelihood-based phylogenetic analyses in the presence of missing data. Cladistics.

[evt195-B92] Singleton R, Amelunxen RE (1973). Proteins from thermophilic microorganisms. Bacteriol Rev..

[evt195-B93] Slonim N, Elemento O, Tavazoie S (2006). Ab initio genotype-phenotype association reveals intrinsic modularity in genetic networks. Mol Syst Biol..

[evt195-B94] Stamatakis A (2006). RAxML-VI-HPC: maximum likelihood-based phylogenetic analyses with thousands of taxa and mixed models. Bioinformatics.

[evt195-B95] Stohr R, Waberski A, Völker H, Tindall BJ, Thomm M (2001). *Hydrogenothermus marinus* gen. nov., sp. nov., a novel thermophilic hydrogen-oxidizing bacterium, recognition of *Calderobacterium hydrogenophilum* as a member of the genus *Hydrogenobacter* and proposal of the reclassification of *Hydrogenobacter acidophilus* as *Hydrogenobaculum acidophilum* gen. nov., comb. nov., in the phylum “Hydrogenobacter/Aquifex”. Int J Syst Evol Microbiol..

[evt195-B96] Sukumaran J, Holder MT (2010). DendroPy: a python library for phylogenetic computing. Bioinformatics.

[evt195-B97] Tatusov RL, Koonin EV, Lipman DJ (1997). A genomic perspective on protein families. Science.

[evt195-B109] Typas A, Banzhaf M, Gross CA, Vollmer W (2011). From the regulation of peptidoglycan synthesis to bacterial growth and morphology. Nat Rev Microbiol..

[evt195-B98] Vollmer W, Bertsche U (2008). Murein (peptidoglycan) structure, architecture and biosynthesis in *Escherichia coli*. Biochim Biophys Acta..

[evt195-B99] Wang X, Quinn PJ (2010). Lipopolysaccharide: biosynthetic pathway and structure modification. Prog Lipid Res..

[evt195-B101] Whelan S, Goldman N (2001). A general empirical model of protein evolution derived from multiple protein families using a maximum-likelihood approach. Mol Biol Evol..

[evt195-B102] Wolf YI, Rogozin IB, Grishin NV, Tatusov RL, Koonin EV (2001). Genome trees constructed using five different approaches suggest new major bacterial clades. BMC Evol Biol..

[evt195-B103] Wu M, Eisen JA (2008). A simple, fast, and accurate method of phylogenomic inference. Genome Biol..

[evt195-B104] Wu D (2009). A phylogeny-driven genomic encyclopaedia of Bacteria and Archaea. Nature.

[evt195-B105] Yoshida M, Muneyuki E, Hisabori T (2001). ATP synthase—a marvellous rotary engine of the cell. Nat Rev Mol Cell Biol..

[evt195-B106] Zhaxybayeva O (2009). On the chimeric nature, thermophilic origin, and phylogenetic placement of the Thermotogales. Proc Natl Acad Sci U S A..

