# Supplementary material for: Being Aquifex aeolicus: Untangling a Hyperthermophile’s Checkered Past
Source: Genome Biol Evol. 2013 Nov 26;5(12):2478–97. doi: 10.1093/gbe/evt195 (PMC3879981; doi:10.1093/gbe/evt195)
Supplement: Supplementary Data [file supp_5_12_2478__index.html]

Being Aquifex aeolicus: Untangling a Hyperthermophile's Checkered Past — Being Aquifex aeolicus: Untangling a Hyperthermophile’s Checkered Past — Supplementary Data 

# Being *Aquifex aeolicus*: Untangling a Hyperthermophile’s Checkered Past

## Supplementary Data

files

**Files in this Data Supplement:**

- Supplementary Data - pdf file
