# Supplementary material for: Being Aquifex aeolicus: Untangling a Hyperthermophile’s Checkered Past
Source: Genome Biol Evol. 2013 Nov 26;5(12):2478–97. doi: 10.1093/gbe/evt195 (PMC3879981; doi:10.1093/gbe/evt195)
Supplement: Supplementary Data [file supp_evt195_SuppMat.pdf]

Table 1. Functional breakdown of all child categories of a) Cellular processes and signaling, b) Information storage and processing, c) Metabolism and d) poorly characterized.

Functional Category	RET	ET	RE	RT	R	E	T	Aq-only	Other	Total
Cell cycle control, cell division, chromosome partitioning (D)	7	3	0	1	0	2	1	1	1	16
Cell wall/membrane/envelope biogenesis (M)	36	15	8	1	3	21	1	7	12	104
Cell motility (N)	6	22	0	0	0	5	2	18	3	56
Posttranslational modification, protein turnover, chaperones (O)	23	8	13	7	11	4	2	7	13	88
Signal transduction mechanisms (T)	13	3	7	0	4	3	1	2	8	41
Intracellular trafficking, secretion, and vesicular transport (U)	4	17	3	1	0	7	1	13	7	53
Defense mechanisms (V)	5	1	2	1	0	0	0	0	1	10
Extracellular structures (W)	0	0	0	0	0	0	0	0	0	0
Nuclear structure (Y)	0	0	0	0	0	0	0	0	0	0
Cytoskeleton (Z)	0	0	0	0	0	0	0	0	0	0

a)

Functional Category	RET	ET	RE	RT	R	E	T	Aq-only	Other	Total
RNA processing and modification (A)	0	0	0	0	1	0	0	0	0	1
Chromatin structure and dynamics (B)	1	0	0	0	0	0	0	0	0	1
Translation, ribosomal structure and biogenesis (J)	57	40	2	3	2	3	7	19	3	136
Transcription (K)	11	11	8	3	3	2	2	5	6	51
Replication, recombination and repair (L)	35	15	6	12	12	5	3	10	9	107

b)

Functional Category	RET	ET	RE	RT	R	E	T	Aq-only	Other	Total
Energy production and conversion (C)	39	0	25	5	12	14	0	10	8	113
Amino acid transport and metabolism (E)	72	1	12	4	7	2	0	2	3	103
Nucleotide transport and metabolism (F)	30	1	8	5	1	0	2	1	0	48
Carbohydrate transport and metabolism (G)	27	2	9	7	6	2	1	2	3	59
Coenzyme transport and metabolism (H)	43	6	15	5	7	2	0	2	4	84
Lipid transport and metabolism (I)	9	13	2	2	4	7	0	1	4	42
Inorganic ion transport and metabolism (P)	28	0	24	2	10	8	0	2	2	76
Secondary metabolites biosynthesis, transport and catabolism (Q)	5	2	8	1	2	4	0	3	2	27

c)

Functional Category	RET	ET	RE	RT	R	E	T	Aq-only	Other	Total
General function prediction only (R)	47	11	39	17	28	11	8	13	25	199
Function unknown (S)	13	7	14	5	27	17	9	12	22	126
Unassigned COG function (Unassigned)	2	2	6	2	22	60	4	683	111	892

d)
